# Pharmacogenetics in Primary Headache Disorders

**DOI:** 10.3389/fphar.2021.820214

**Published:** 2022-02-10

**Authors:** Irina I. Belyaeva, Anna G. Subbotina, Ivan I. Eremenko, Vadim V. Tarasov, Vladimir N. Chubarev, Helgi B. Schiöth, Jessica Mwinyi

**Affiliations:** ^1^ Department of Surgical Sciences, Functional Pharmacology and Neuroscience, University of Uppsala, Uppsala, Sweden; ^2^ Department of Pharmacology, Institute of Pharmacy, I. M. Sechenov First Moscow State Medical University, Moscow, Russia; ^3^ Institute of Translational Medicine and Biotechnology, I. M. Sechenov First Moscow State Medical University, Moscow, Russia

**Keywords:** pharmacogenetics, migraine, cluster headache (CH), tension-type headache, drug treatment, pharmacokinetics, pharmacodynamics

## Abstract

Primary headache disorders, such as migraine, tension-type headache (TTH), and cluster headache, belong to the most common neurological disorders affecting a high percentage of people worldwide. Headache induces a high burden for the affected individuals on the personal level, with a strong impact on life quality, daily life management, and causes immense costs for the healthcare systems. Although a relatively broad spectrum of different pharmacological classes for the treatment of headache disorders are available, treatment effectiveness is often limited by high variances in therapy responses. Genetic variants can influence the individual treatment success by influencing pharmacokinetics or pharmacodynamics of the therapeutic as investigated in the research field of pharmacogenetics. This review summarizes the current knowledge on important primary headache disorders, including migraine, TTH, and cluster headache. We also summarize current acute and preventive treatment options for the three headache disorders based on drug classes and compounds taking important therapy guidelines into consideration. Importantly, the work summarizes and discusses the role of genetic polymorphisms regarding their impact on metabolism safety and the effect of therapeutics that are used to treat migraine, cluster headache, and TTH exploring drug classes such as nonsteroidal anti-inflammatory drugs, triptans, antidepressants, anticonvulsants, calcium channel blockers, drugs with effect on the renin–angiotensin system, and novel headache therapeutics such as ditans, anti-calcitonin-gene-related peptide antibodies, and gepants. Genetic variants in important phase I-, II-, and III-associated genes such as cytochrome P450 genes, UGT genes, and different transporter genes are scrutinized as well as variants in genes important for pharmacodynamics and several functions outside the pharmacokinetic and pharmacodynamic spectrum. Finally, the article evaluates the potential and limitations of pharmacogenetic approaches for individual therapy adjustments in headache disorders.

## Introduction

Approximately one-half of the adult population worldwide is affected by a headache disorder (Hainer and Matheson, 2013). Headache is the most frequently occurring neurological symptom and the most prevalent type of pain in children and adolescents (Faedda et al., 2016). Headache has a disabling potential and leads to reduced life quality and limitations in the social sphere (Nieswand et al., 2020). Headache disorders are ranked as the second most common cause of years of life spent with disability worldwide (Schwaiger et al., 2009; Saylor and Steiner, 2018). For tension-type headache (TTH), the global age-standardized prevalence was 26.1% overall: 30.8% for women and 21.4% for men, and for migraine, the global age-standardized prevalence was 14.4% overall: 18.9% for women and 9.8% for men, according to the Global Burden of Diseases, Injuries, and Risk Factors studies (GBD 2016 Headache Collaborators, 2018). In the European Union, the total annual cost of headache among adults aged 18–65 years was estimated at 173 billion euros, apportioned in 64% to migraine, 12% to TTH, 21% to medication-overuse headache, and 2% to other types of headaches (Linde et al., 2012).

Today, a high number of treatment possibilities are available for headache disorders. However, headache patients react differently to given drugs leading to a wide variety of treatment responses. It is estimated that only 50% of migraine patients adequately respond to acute and preventive therapy (Pomes et al., 2019; Zobdeh et al., 2021). Likewise, many patients with cluster headache fail to respond adequately to pharmacological treatment approaches. Additionally, a significant number of these patients experience misdiagnoses or diagnostic delays, which constraints the possibility of the timely application of adequate abortive and preventive therapy (Ljubisavljevic and Zidverc Trajkovic, 2019). The therapeutic efficacy of analgesics in TTH tends to decrease with increasing headache frequency (Bendtsen et al., 2010). Especially, patients with TTH have a higher risk of developing medication overuse headache due to the regular and frequent use of analgesics, with a subsequent drug-induced increase in headache frequency and chronification (Diener et al., 2019).

Pharmacogenetics investigates the association between genetic markers and the pharmacokinetics and pharmacodynamics of the drug and aims at predicting the individual therapy response based on these associations (Valdes and Yin, 2016). In recent times, advances have been made in neuropsychiatry regarding the understanding of the role of genetic polymorphisms in therapy response to treatment approaches as in major depressive disorder (Shalimova et al., 2021), schizophrenia (Jovanović et al., 2010), or epilepsy (Al-Eitan et al., 2020). The role of pharmacogenetics for therapy success with compounds used for the treatment of headache disorders is not as largely investigated, and pharmacogenetic information is hitherto practically not used to predict efficacy or adverse effects in clinical practice in this field (Christensen et al., 2016).

A rather broad number of therapeutics are nowadays available for the treatment of headache disorders. These include, e.g., nonsteroidal anti-inflammatory drugs (NSAIDs), triptans, ergot derivatives, gepants, and ditans for the acute treatment for migraines and calcitonin-gene-related peptide (CGRP) monoclonal antibodies, onabotulinumtoxinA, calcium channel blockers (CCBs), beta-blockers, and anticonvulsants for prophylactic treatment for migraines. Cluster headache is as well treated by triptans and ergot derivatives besides the use of oxygen in the acute phase and by anti-CGRP monoclonal antibodies, CCBs, anticonvulsants prophylactically. NSAIDs are used as an acute treatment for TTH. Tricyclic antidepressants (TCAs) and neurotransmitter release antagonists are used as prophylactic treatment for TTH. Therapeutics of several important classes of drugs that are used for the treatment of headache disorders have been shown to be substrates of polymorphically expressed metabolizing enzymes, transport systems, or receptors. However, the translation of this knowledge into applicable recommendations for individual dose adjustments is currently missing in the area of headache disorders.

The review summarizes the current knowledge about drug classes and compounds used for the treatment of commonly occurring and important types of primary headache disorders, characterizes important pharmacogenetic variants that affect the pharmacokinetics and pharmacodynamics of these drugs, and sheds light on the question to what extent the pharmacogenetic achievements may have been translated into treatment recommendations.

## Methods

The review aimed to include broad and comprehensive information regarding treatment guidelines and pathophysiology of the headache disorders TTH, cluster headache, and migraine and information regarding pharmacogenetics and its role in the safety and efficacy of drugs used for the treatment of the mentioned diseases. This was achieved by a thorough search in the scientific literature using the search database PubMed and using search terms in different combinations and different long combination chains including “epidemiology, prevalence of migraine/TTH/cluster headache; pathophysiology, triggers, symptoms, diagnosis, acute/chronic treatment, treatment recommendations, pharmacotherapy of migraine/TTH/cluster headache; mechanism of action, metabolism/metabolic pathways, pharmacokinetics, pharmacodynamics, pharmacogenetics, candidate genes, genetic polymorphism/SNP/genetic variants, cytochrome P450/CYP, safety, efficacy/effect, adverse effects/adverse reactions in combinations which each of the headache disorders and different recommended drug/drug classes.” The focus was mainly laid on drug and drug classes recommended by the official treatment guidelines in Europe and the USA, which were considered separately in the search runs together with the three different headache types in focus. Articles considered were mainly clinical trials and studies that included human subjects.

## Important Types of Headaches—Disease Burden, Clinical Forms, and Pathophysiology

### Migraine

Migraine is one of the most common types of headaches. Twelve percent are affected at least once by an acute migraine attack, and approximately 2.5% of the global population show chronic migraine. Migraine ranks second place among the causes of temporary disability worldwide (GBD 2016 Headache Collaborators, 2018; Burch et al., 2019). Migraine is characterized by a strong unilateral headache occurring with nausea, vomiting, photophobia, and phonophobia that lasts from several hours to several days. Two main subtypes of migraine are distinguished, i.e., migraine with aura and migraine without aura (Cephalalgia, 2018). In contrast to migraine without aura, migraine with aura manifests with a headache accompanied by various local neurological and visual symptoms, of which photopsia, photophobia, and temporary visual disturbances are very common. Symptoms, such as, e.g., sensitivity disorders, vestibular symptoms, or temporary paresis, may also be observed. Rare and atypical migraine syndromes include familial hemiplegic migraine, basilar migraine, ophthalmoplegic migraine, and exceptional retinal migraine (Fraser et al., 2019). In contrast to episodic migraine, chronic migraine occurs on 15 or more days per month for at least 3 months (Cephalalgia, 2018).

The etiopathogenetic mechanisms of migraine development are not fully understood. Migraine affects women three times more often than men (Broner et al., 2017) and shows the highest incidences in young patients between 30 and 40 years (GBD 2016 Headache Collaborators, 2018). There is evidence for a hereditary component that influences the likelihood of developing migraine (de Boer et al., 2019). Genetic susceptibility factors differ between migraine with and without aura (Pisanu et al., 2017a). Other risk factors comprise concomitant psychological and psychiatric disorders (Pisanu et al., 2020), hormonal status, myofascial syndromes, and the influence of various adverse environmental factors, including nutritional factors or stressful situations (Chai et al., 2014; Pisanu et al., 2017b; Marmura, 2018; Zobdeh et al., 2021). Numerous studies have identified a variety of factors that can trigger migraine attacks. Examples are psychological and physical stress, menstrual cycle changes, weather changes, sleep disturbances, alcohol and other dietary compounds, barometric changes, or starvation (Marmura, 2018). The widely accepted theory of migraine pathophysiology suggests the abundance of a neurovascular conflict involving the trigeminal nerve and underlying neuroinflammation, which causes changes in vascular tone, represented by initial vasospasm and subsequent vasodilation (Charles, 2018; Ferrari et al., 2015; Kojić and Stojanović, 2013). It is assumed that the trigeminovascular conflict is preceded by complex interactions between the cortex, hypothalamus, and subcortical nuclei responsible for pain perception and activation of the antinociceptive system (Dodick, 2018). The interaction between the hypothalamus and the trigeminal nucleus caudalis probably triggers the activation of the trigeminovascular system and subsequently occurring migraine attacks. Cortical spreading depression is assumed to play a key role in the trigeminal activation in migraine with aura (Haanes and Edvinsson, 2019).

An imbalance in the system of serotonin and norepinephrine and other monoamines is assumed to also play a role in the pathogenesis of migraine (Tardiolo et al., 2019). In recent years, CGRP has been recognized as one of the key mediators of migraine development. CGRP plays a relevant role in the trigeminovascular system as an intermediary agent between vascular reactions and the perception of changes in vascular tone as a pain stimulus (Iyengar et al., 2019). It has been shown that CGRP levels are significantly increased in migraine patients compared with the healthy population (Schou et al., 2017). CGRP release from the primary afferent fibers in the trigeminal ganglion is closely associated with the nitric oxide system and other agents that cause peripheral and central neuronal sensitization, which underlies the transition of acute to chronic forms of migraine (Goadsby et al., 2017; Puledda et al., 2017). The elucidation of CGRP led to a new era in migraine therapy based on CGRP antibodies and receptor antagonists that are currently the most efficient compounds available in chronic migraine (Deen et al., 2017; Negro and Martelletti, 2019).

### Tension-Type Headache

TTH affects many individuals worldwide (Ashina et al., 2021). The lifetime prevalence of TTH ranges between 30 and 78% (Wrobel Goldberg et al., 2014). According to the European Headache Federation (EHF), most people occasionally experience TTH, whereas 10% show frequent repeats of TTH. Chronic TTH occurs in approximately 3% of adults and in some children in Europe (Steiner et al., 2019). Mainly because of its high prevalence, the socioeconomic consequences of TTH are significant (Wrobel Goldberg et al., 2014). Studies show that frequent episodic and chronic TTH occur significantly more frequently in women than men (Russell, 2005). TTH is most commonly observed among women aged 15–49 years (GBD 2016 Headache Collaborators, 2018).

TTH is subdivided into episodic and chronic types. Although infrequent episodic TTH usually has a rather little impact on the individual and is often self-limiting, frequent episodic TTH can be associated with considerable disability and may warrant pharmacological treatment (Cephalalgia, 2018). Chronic TTH occurs on 15 days a month or more for at least 3 months, may be daily and unremitting, and may be associated with mild nausea (Steiner et al., 2019). Chronic TTH is a serious disease, causing greatly decreased quality of life and a high rate of disability (Cephalalgia, 2018).

The pathophysiology of TTH is still not fully understood (Monteith and Oshinsky, 2009). Sensitization of second-order neurons at the level of the spinal dorsal horn or trigeminal nucleus, sensitization of supraspinal neurons, and decreased descending inhibition from supraspinal structures play a major role in the pathophysiology of chronic TTH. Furthermore, an increased pericranial myofascial pain sensitivity may also play a role (Bendtsen, 2000). The pain is usually bilateral, mild to moderate, is of a pressing or tightening quality, and is usually not accompanied by other symptoms (Wrobel Goldberg et al., 2014). Sometimes, it is difficult to distinguish between migraine, TTH, or other headache types (Burch, 2019a). Several triggers are known for TTH. Certain myofascial trigger points in the muscles of the head and neck may cause a local twitching reaction and spontaneous pain (Gildir et al., 2019). Sleep disorders have been identified as a risk factor for TTH and a transition from episodic to chronic TTH (Rains et al., 2015). TTH can also be triggered by negative affect and sunlight (Wang et al., 2013). Frequent episodic and chronic TTH is assumed to be caused by a combination of genetic and environmental factors, whereas infrequent episodic TTH is assumed to be primarily triggered by environmental factors (Russell, 2007).

TTH therapy comprises both non-pharmacological treatments, including patient education, disease information, and lifestyle change advice, and pharmacological therapy approaches, such as the use of NSAIDs and TCAs (Lenaerts, 2009) (Tables 1 and 2). The choice of a particular approach depends on the severity of the disease. Mild forms should be initially non-pharmacologically treated (Steiner et al., 2019). Amitriptyline may be used as a preventive therapy for chronic TTH (Wrobel Goldberg et al., 2014). Nonprescription analgesics are indicated for the management of episodic TTH (Scripter, 2018). Chronic TTH often does not respond to drug treatment and makes it necessary to apply psychological approaches (Steiner et al., 2019).

**TABLE 1:** Treatment principles of primary headache disorders—acute treatment options.

Headache type	Drug group for treatment	Examples of drugs	Target molecule(s)	Adverse effects (example)s
Migraine and TTH	Antiplatelet agents Loder et al. (2012), Marmura et al. (2015), Orr et al. (2015), Steiner et al. (2019)	Aspirin Diener (1999), Lange et al. (2000), Farinelli and Martelletti, (2007), Lenaerts, (2009), Marmura et al. (2015), Derry et al. (2017)	COX-1, COX-2 Farinelli and Martelletti, (2007)	Tiredness nausea, risk of aspirin-induced asthma, nephrotoxicity long-term Graham and Scott, (2005), Kim et al. (2010a), Marjan et al. (2014)
Migraine and TTH	Nonopioid analgesics Mayans and Walling, (2018), Steiner et al. (2019)	Acetaminophen Lipton et al. (2000), Marmura et al. (2015)	COX-1, COX-2 Blanco et al. (2008), McEvoy et al. (2021)	Hepatotoxicity allergic reactions hematotoxicity Hinson et al. (2010), Zajic et al. (2019)
Migraine and TTH	NSAIDs Steiner et al. (2019), Marmura et al. (2015), Pardutz and Schoenen, (2010), National Clinical Guideline Centre (UK), 2012)	Diclofenac Marmura et al. (2015), Joshi and Rapoport, (2017), McVige et al. (2020)	COX-1, COX-2 Pardutz and Schoenen, (2010)	Gastrointestinal adverse effects (bleeding) Daly et al. (2007), Blanco et al. (2008), Figueiras et al. (2016) liver failure, water and sodium retention Makar et al. (2013) colon and rectal cancer,Makar et al. (2013)
		Naproxen (Yang (2013), Marmura et al. (2015)		
		Ibuprofen Derry et al. (2015), Marmura et al. (2015), Pavithra et al. (2020)		
		Ketorolac Marmura et al. (2015)	COX-1	
		Ketoprofen Dib et al. (2002), Marmura et al. (2015)		
Migraine	Opioid analgesics Marmura et al. (2015), Owusu Obeng et al. (2017), Mayans and Walling, (2018)	Butorphanol Hoffert et al. (1995), Rapoport et al. (2004), Marmura et al. (2015)	ϰ-opioid receptor (agonist)	Addiction, sedation, dizziness, nausea Deen et al. (2017) inhibition of respiratory activity and cough Ho et al. (2008)
			μ-opioid receptor (antagonist) Ho et al. (2008)	
—	—	Tramadol Alemdar et al. (2007), Marmura et al. (2015)	μ-opioid receptor (agonist) Gong et al. (2014)	Nausea, vomiting dizziness, headache hypotension Tzvetkov et al. (2011)
Migraine	Gepants Deen et al. (2017), Tepper, (2018), Negro and Martelletti, (2019), Steiner et al. (2019), de Vries et al. (2020), Moreno-Ajona et al. (2020)	Telcagepant Ho et al. (2008), Digre, (2019)	CGRP receptor (RAMP1) Digre, (2019)	Hepatotoxicity Szkutnik-Fiedler, (2020)
		Ubrogepant Digre, (2019)		
		Rimegepant,Digre, (2019)		
		Atogepant		
Migraine	Ditans Steiner et al. (2019)	Lasmiditan Szkutnik-Fiedler, (2020)	5-HTR_1F_ Vila-Pueyo, (2018)	Paresthesia, dizziness nausea de Vries et al. (2020)
Migraine Cluster headache	Ergot derivatives Marmura et al. (2015), Robbins et al. (2016), Steiner et al. (2019)	Ergotamine Dahlöf and Maassen Van Den Brink, (2012), Marmura et al. (2015), Mayans and Walling, (2018)	5-HT1B, 5-HT1D, 5-HT1F Diener and Limmroth, (2001)	Peripheral vasoconstriction Roberto et al. (2015), Antonaci et al. (2016), risk of ischemic complications Wammes-van der Heijden et al. (2006), Antonaci et al. (2016), nausea Magnoux and Zlotnik (2004), Dahlöf and Maassen Van Den Brink (2012), ergotism Srisuma et al. (2014)
		Dihydroergotamine Schiff, (2006), Dahlöf and Maassen Van Den Brink, (2012)		
Migraine and Cluster headache	Triptans Tfelt-Hansen et al. (2000), Marmura et al. (2015), Robbins et al. (2016), Mayans and Walling, (2018), Rizzoli, (2018), Steiner et al. (2019)	Sumatriptan Moskowitz and Cutrer, (1993), Marmura et al. (2015)	5-HTR_1B/1D_ Ramírez Rosas et al. (2013)	Coronary vasospasm, myocardial ischemia, arrhythmias, peripheral vasospasm, nausea and vomiting Vila-Pueyo, (2018)
		Zolmitriptan Marmura et al. (2015)		
		Eletriptan Marmura et al. (2015)		
		Naratriptan, Marmura et al. (2015)		
Cluster headache	—	Oxygen Kudrow, (1981), Fogan, (1985), Cohen et al. (2009), Robbins et al. (2016)	Neurons in trigeminocervical complex	—

COX: Cyclooxygenase; NSAIDs: Nonsteroidal anti-inflammatory drugs; CGRP: Calcitonin-gene related peptide; 5-HTR: Serotonin receptor; TTH: Tension-type headache.

**TABLE 2:** Treatment principles of primary headache disorders—treatment options for prevention.

Headache type	Drug group for treatment	Examples of drugs	Target molecule(s)	Adverse effects (examples)
Migraine and TTH	Antidepressants Burch, (2019b), Steiner et al. (2019)	Nortriptyline Steiner et al. (2019)	Blocks uptake of serotonin and norepinephrine	Sedation, dry mouth, weight gain, constipation Ha and Gonzalez, (2019), urinary retention
		Amitriptyline Bendtsen et al. (2010), Hicks et al. (2013), Palacios-Ceña et al. (2019)		
Migraine	—	Venlafaxine Johnson et al. (2006), Dean et al. (2015)	Blocks uptake of serotonin and norepinephrine	Hypertension, dry mouth, insomnia, nausea Ha and Gonzalez, (2019), sweating, weight loss, asthenia, sexual dysfunction tachycardia Johnson et al. (2006)
Migraine	—	Duloxetine Volpe, (2008), Young et al. (2013)	Blocks uptake of serotonin and norepinephrine	Headache, gastrointestinal adverse effects, dizziness, sleep disturbances, sexual dysfunction
TTH	—	Mirtazapine Dahl et al. (1997), Bendtsen and Jensen, (2004), Lavergne et al. (2005)	α2- receptors, 5HT-2, 5HT-3, H1- receptors Leung, (2021)	Xerostomia, constipation, sedation, confusion Lenaerts, (2009)
Migraine	CGRP monoclonal antibodies de Vries et al. (2020), Scuteri et al. (2021)	Erenumab Sacco et al. (2019) ^1^	CGRP receptor	Headache, skin reactions, joint pain
—	—	Fremanezumab Sacco et al. (2019), Urits et al. (2020)	CGRP	Pain, induration, and erythema at site of injection, fatigue, nausea, nasopharyngitis headache hematuria, contact dermatitis diarrhea, increased ALT Szkutnik-Fiedler, (2020)
—	—	Eptinezumab Sacco et al. (2019), Lipton et al. (2020)	CGRP	
Migraine and Cluster Headache	—	Galcanezumab Company ELa, (2019), Sacco et al. (2019)	CGRP	
Migraine	Beta-blockers Ha and Gonzalez, (2019), Steiner et al. (2019)	Atenolol Silberstein et al. (2012), Steiner et al. (2019)Metoprolol (Silberstein et al., 2012, Steiner et al. (2019)Propranolol Silberstein et al. (2012), Stovner et al. (2014), Steiner et al. (2019)	β-receptors Fumagalli et al. (2020)	Bradycardia hypotension, fatigue, gastrointestinal adverse effects, reduction of GFR Ha and Gonzalez, (2019)
Migraine	Calcium channel blockers Robbins et al. (2016), McKeever and Hamilton, (2021)	Flunarizine Steiner et al. (2019), Stubberud et al. (2019)	Ca + channel McKeever and Hamilton, (2021)	Constipation bradycardia, hypotension peripheral oedema and impotence fatigue, asthenia, weight increase Aurora et al. (2011)
—	—	Cinnarizine (Silberstein, 1996)	—	Extrapyramidal reactions, depression in elderly patients Taghdiri et al. (2014)
Migraine and Cluster Headache	—	Verapamil Leone et al. (2000), Diener et al. (2010), Francis et al. (2010), Steiner et al. (2019), Tso et al. (2021)	Ca + channel McKeever and Hamilton, (2021)	Hypotension, bradycardia, AV block, constipation, arrhythmia, fatigue, bradycardia Pahor et al. (1996), Leone et al. (2000), Silberstein, (2000), Brandt et al. (2020)
Migraine	ARBs Halker et al. (2016)	Candesartan Stovner et al. (2014), Halker et al. (2016), Sánchez-Rodríguez et al. (2021)	Angiotensin II receptor Tronvik et al. (2006)	Respiratory tract infections sleep problems Heidari et al. (2017)
Migraine	ACE inhibitors Halker et al. (2016)	Lisinopril Schrader et al. (2001)	ACE Tronvik et al. (2006)	Coughing dizziness
Migraine	Neurotransmitter release antagonists Herd et al. (2018), Affatato et al. (2021)	OnabotulinumtoxinA Binder et al. (2000), Erdemoglu and Varlibas, (2007), Diener et al. (2010), Aurora et al. (2011)	Transport protein SNAP-25	Muscular weakness musculoskeletal pain neck pain
Migraine	Anticonvulsants (Steiner et al., 2019)	Valproate sodium/Divalproex sodium Hering and Kuritzky (1989), Silberstein, (1996), Gallagher et al. (2002), Lovell and Marmura, (2010), Marmura et al. (2015), Ha and Gonzalez, (2019)	Na + -K channels, GABA transaminase Ito et al. (1990), Van den Berg et al. (1993), Johannessen and Johannessen, (2003)	Gastrointestinal adverse effects (nausea, anorexia, diarrhea), tremor, weight gain, sedation Ha and Gonzalez (2019)
Migraine and Cluster headache	—	Topiramate (Garnett, 2000, Gryder and Rogawski, (2003), Diener et al. (2009), Ha and Gonzalez, (2019)	Na + channel AMPA receptors Doose et al. (1996), Fan et al. (2016)	Paresthesia, Ha and Gonzalez, (2019) upper respiratory tract infection, fatigue Johannessen, (1997)
Migraine	—	Phenytoin Mamiya et al. (1998)	Na + -K + -ATPase Depondt et al. (2011)	Ataxia, nystagmus, nausea, tremor encephalopathy Kidd et al. (2001), Azzato et al. (2010), Depondt et al. (2011), Babu et al. (2013), Marmura and Kumpinsky, (2018)
Migraine	—	Carbamazepine US Food and Drug Administration, (2007)	Na + channel	Memory problems, ataxia, diplopia, rash Hung et al. (2006), Man et al., 20072008, US Food and Drug Administration, (2007), Ikeda et al. (2010)
Cluster Headache	—	Steroid injection of GON Gantenbein et al. (2012), Robbins et al. (2016)	Steroid receptors Watanabe and Bruera, (1994), Afridi et al. (2006), Robbins et al. (2016), Gaul et al. (2017)	Local pain steroid effects (facial oedema, sleeping disorders, acne) bradycardia Gantenbein et al. (2012)

5-HTR: Serotonin receptor; CGRP: Calcitonin-gene related peptide; GFR: Glomerular filtration rate; AV: Atrioventricular; ARB: Angiotensin II, receptor blocker; ACE: Angiotensin-converting enzyme; SNAP-25: Synaptosomal-associated protein, 25 kDa; GABA: Gamma-aminobutyric acid; AMPA: α-amino-3-hydroxy-5-methyl-4-isoxazole propionic acid; ATPase: Adenosine 5′-triphosphatase; GON: Greater occipital nerve; TTH: Tension-type headache.

### Cluster Headache

Cluster headache has a prevalence of 0.2–0.3% in Europe, and it has a rather low frequency compared with other headaches considered in this review (Stovner and Andree, 2010). The headache type is more prevalent in men with a male-to-female ratio of 6–7:1 (Rozen et al., 2001) and typically begins between 20 and 40 years of age (Weaver-Agostoni, 2013). The severity of the disorder has major effects on the patient's quality of life and is, in some very severe cases, associated with suicidal ideation (Hoffmann and May, 2018). Patients describe a cluster headache attack as an intense and severe strictly unilateral pain, typically in the supraorbital, retro-orbital, or temporal regions and arising from deep within (Wei et al., 2019). Attacks last 15–180 min and may be accompanied by tears, conjunctival injection (redness of the sclera), rhinorrhea, nasal congestion, hyperhidrosis (excessive sweating), and eyelid edema (May et al., 2018). Cluster headache attacks can occur once every other day at the start of the disease and increase up to eight times a day (Lai et al., 2009). Cluster headache is subdivided into episodic and chronic types. Patients are deemed to have episodic cluster headache if the break between the bouts lasts longer than 3 months while not taking a preventive treatment. Patients who have bouts of attacks with breaks of less than 3 months between are classified as having chronic cluster headaches (Cephalalgia, 2018).

The exact pathophysiology of cluster headache remains unknown (Brandt et al., 2020). Current theories implicate mechanisms, such as vascular dilation, trigeminal nerve stimulation, and circadian effects, histamine release, genetic factors, and autonomic nervous system activation (Weaver-Agostoni, 2013). Triggers for cluster headaches include vasodilators (e.g., alcohol, nitroglycerin) and histamine. Tobacco use or secondary childhood exposure is a risk factor for cluster headaches (Weaver-Agostoni, 2013). There is, to some degree, a genetic component, as first-degree family members are 18 times more likely to be diagnosed with cluster headaches (Kandel and Mandiga, 2021).

Cluster headache requires drug therapy (Weatherall, 2015). The usual first-line preventive treatment for cluster headache is verapamil (Weatherall, 2015; Leone et al., 2000) (Table 2). If verapamil is not tolerated or effective, then typical second-line therapies include topiramate and lithium carbonate (Weatherall, 2015). For acute treatment of cluster headache, triptans, high-flow oxygen, octreotide, or local anesthetics are used (Cohen et al., 2009; Law et al., 2013; Weatherall, 2015). Effective treatment of cluster headache should be established by a doctor as soon as possible. In addition to drug therapy, neuromodulation can be used. Every patient with active cluster headache requires frequent follow-up both to ensure that optimum acute and preventative treatments are maintained and to monitor for treatment toxicity (Steiner et al., 2019).

## Important Drug Groups Used for the Treatment of Headache Disorders

### Migraine

Migraine treatment can be divided into two main areas, i.e., the treatment of an acute migraine attack to achieve symptomatic pain relief and pharmacological prevention of attacks in chronic migraine (Digre, 2019). For relief of an acute migraine attack, EHF, the American Academy of Neurology (AAN), and the Canadian Headache Society recommend using drugs belonging to the drug classes of triptans (sumatriptan, zolmitriptan, eletriptan, and naratriptan), NSAIDs (ibuprofen, ketoprofen, naproxen, diclofenac, and ketorolac), aspirin, paracetamol, and gepants (telcagepant, ubrogepant, rimegepant, and atogepant), and ditans (lasmiditan) (Loder et al., 2012; Marmura et al., 2015; Orr et al., 2015; Rawson et al., 2017; Steiner et al., 2019). In very difficult cases with uncontrolled symptoms, opioids, such as tramadol or butorphanol, can be used according to AAN and American Headache Society (AHS) (Loder et al., 2012; Marmura et al., 2015). Treatment choice should be based on the patient's age, the presence of concomitant diseases, and the effectiveness in the particular patient (Rizzoli, 2018). Most of these therapeutics are only effective in relieving the pain symptoms of migraine but are not effective against aura symptoms. Triptans have been shown to improve also symptoms such as photopsia and visual disturbances (Tang et al., 2020). Ergot alkaloids remain as second-line options due to their adverse event profile with increased risk, e.g., cardiovascular adverse effects (Tfelt-Hansen et al., 2000; Dahlöf and Maassen Van Den Brink, 2012; Srisuma et al., 2014; Antonaci et al., 2016; Mayans and Walling, 2018).

Drugs used for the treatment of acute migraine have relevant adverse effects. NSAIDs are associated with gastrointestinal bleeding, hepatotoxicity, and cardiovascular adverse effects, whereas the most important adverse effects for acetaminophen are hepatotoxicity, allergic reactions, and hematotoxicity (Zajic et al., 2019). Gepants (ubrogepant and rimegepant) are known to be associated with hypersensitivity reactions, nausea, insomnia, dry mouth, and probably hepatotoxicity (Moreno-Ajona et al., 2020; Szkutnik-Fiedler, 2020). Triptans can induce peripheral vasospasm, coronary vasospasm, myocardial ischemia, arrhythmias, nausea, and vomiting, whereas ditans can cause sedation, vertigo, paraesthesia, and anxiety (Vila-Pueyo, 2018) (Table 1).

Preventive treatment of migraines can be divided into three main areas, i.e., preemptive, short-term, and maintenance treatment (Sacco et al., 2019). Preemptive treatment is relevant in the case a trigger is known (e.g., physical activity). In this case, a drug such as, e.g., an NSAID or triptan, is taken a few hours before the trigger starts. Short-term treatment can be effective in the case a trigger lasts longer (for example, staying at a high altitude or menstruation) and is implemented through preventive administration of NSAIDs or triptans (frovatriptan) before the start and during the triggering event. Maintenance treatment is carried out constantly and is indicated for frequent migraine attacks that limit the ability to work and reduce the quality of life and for special types of migraines (hemiplegic migraine, migraine with a stem aura) (Ha and Gonzalez, 2019; Marmura et al., 2015). According to the recommendations of EHF, AAN, and the Canadian Headache Society, the key drugs for preventive maintenance treatment include anticonvulsants (sodium valproate, topiramate, and divalproex sodium), drugs from the group of beta-blockers (metoprolol, timolol, propranolol, etc.), TCAs (e.g., amitriptyline), selective serotonin reuptake inhibitors or serotonin–norepinephrine reuptake inhibitors (SNRIs, e.g., venlafaxine), angiotensin-converting enzyme inhibitors (ACE inhibitors, lisinopril), angiotensin receptor blockers (ARBs, candesartan), and CCBs (e.g., flunarizine and verapamil) (Steiner et al., 2019; Silberstein et al., 2012; Loder et al., 2012) (Table 2). Cinnarizine and verapamil may be used as alternative drugs to prevent migraines, e.g., in refractory migraine cases or when flunarizine is not available (Knezevic et al., 2018). Food and Drug Administration (FDA) (2019) and European Medicines Agency (2018) have recently approved using CGRP monoclonal antibodies (erenumab, galcanezumab, fremanezumab, and eptinezumab) for the prevention treatment of chronic migraine (Steiner et al., 2019; Moreno-Ajona et al., 2020; Sacco et al., 2019; Suwała et al., 2019; Shams et al., 2006). Therapy with botulinum toxin type A (onabotulinumtoxinA) is recommended by EHF and AAN as an equally significant and effective alternative for migraine prevention (Digre, 2019; Affatato et al., 2021; Herd et al., 2018) (Table 2). The drugs described earlier have a wide range of adverse effects. Anticonvulsants are known to be associated with central (vertigo, hallucinations, depression, nystagmus, tinnitus, dysarthria, dyskinesia, etc.) and peripheral (arrhythmias, hematologic toxicity, liver failure, interstitial nephritis, and allergic reactions) adverse effects and have a teratogenic potential (Silberstein, 1996; Diener et al., 2009; Fricke-Galindo et al., 2018b; Marmura and Kumpinsky, 2018). Beta-blockers, calcium antagonists, and ACE inhibitors have been shown to affect the heart rate (bradycardia) and myocardial contractility, blood pressure (hypotension), and renal function (reduction of GFR), respectively (Ha and Gonzalez, 2019; Steiner et al., 2019). Anti-CGRP monoclonal antibodies can cause hypersensitivity reactions (including skin rash, pruritus, angioedema, and anaphylaxis) and constipation (Szkutnik-Fiedler, 2020).

### Tension-Type Headache

The EHF states that the objective of management in both episodic and chronic subtypes of TTH is total attack suppression. Drugs exist to control TTH attacks, but prevention is the overall aim of treatment (Steiner et al., 2019). The EHF recommends starting the treatment of TTH with lifestyle changes and over-the-counter analgesics. If this treatment approach is insufficient amitriptyline or nortriptyline (first line drugs), mirtazapine (second-line drug) or venlafaxine (third-line drug) can be used (Steiner et al., 2019).

A systematic review recently summarized the effectiveness of onabotulinumtoxinA in chronic TTH. The review showed mixed results, which were likely due to variable study designs used in the 22 studies identified, and concluded that there was only low quality of evidence for the effect of onabotulinumtoxinA in TTH (Erdemoglu and Varlibas, 2007). OnabotulinumtoxinA is not recommended for TTH by the EHF and the AAN (Simpson et al., 2016; Steiner et al., 2019). The TCA amitriptyline is widely used for the prophylactic treatment of chronic TTH based on studies that demonstrated the efficacy of amitriptyline in chronic TTH (Ghadiri-Sani and Silver, 2016). Venlafaxine and mirtazapine are also effective for the treatment of TTH (Ashina et al., 2021). The effectiveness of venlafaxine has been proven in a clinical study on 60 patients (Zissis et al., 2007). Mirtazapine's effectiveness in chronic TTH was proven in a clinical trial with 24 patients (Bendtsen and Jensen, 2004). Ibuprofen can be used to treat acute headache attacks in patients with episodic TTH, reaching a relief of paint around 2 h later (Derry et al., 2015) (Tables 1 and 2).

### Cluster Headache

The treatment of cluster headache can be divided into three therapy approaches, i.e., a fast-acting abortive treatment, preventive drug treatment, and transitional treatment approaches to bridge the period between patients starting preventive drug treatment and the drugs asserting an effect. The main goal in cluster headache treatment should always be to prevent all attacks (Brandt et al., 2020).

The EHF recommends triptans and oxygen as an acute treatment of cluster headaches. Preventive treatment should be started using prednisolone as a transition therapy (Obermann et al., 2021) option to obtain a quicker response according to the EHF (Tables 1 and 2). Verapamil, lithium carbonate, and topiramate can be used for maintenance prophylaxis (Steiner et al., 2019). The AHS recommends the use of CCBs (e.g., verapamil) and civamide (capsaicin) and the injection of steroids, lithium, and melatonin as preventive treatment options. The AHS and the EHF recommend triptans and oxygen for acute therapy (Robbins et al., 2016). The EHF and the AHS do not recommend sodium valproate for cluster headaches. Some studies have shown that valproate sodium may be effective for treating cluster headache as reviewed in Magnoux and Zlotnik (2004) and shown for refractory forms of this headache type (Magnoux and Zlotnik, 2004). The effectiveness of ergotamine in cluster headaches varies depending on the expression of certain genes (Borro et al., 2019). According to the AHS, there is insufficient evidence to advise the use of ergotamine for the acute treatment of cluster headaches (Robbins et al., 2016). The EHF does not mention ergotamine as a treatment option for cluster headaches (Steiner et al., 2019). Galcanezumab (Emgality) was recently approved by the FDA for the treatment of episodic cluster headache. The recommended dosage of Emgality is 300 mg (administered as three consecutive subcutaneous injections of 100 mg each) at the onset of the cluster period and then monthly until the end of the cluster period (Company ELa, 2019).

## Pharmacogenetics of Important Drugs Used in Migraine, Tension-Type Headache, and Cluster Headache

### Antiplatelet Agents

Acetylsalicylic acid (ASA, Aspirin) is an irreversible inhibitor of cyclooxygenase-1 (COX-1) and cyclooxygenase-2 (COX-2). The complex effect of aspirin on pain and headache disorders is explained by its anti-inflammatory, antiplatelet, and analgesic properties (Farinelli and Martelletti, 2007). Adverse effects of aspirin are similar to those of NSAIDs. Aspirin is characterized by an increased risk of bleeding (due to antiplatelet properties), a higher risk of aspirin-induced asthma, and rebound headaches with long time use (Marjan et al., 2014; Graham and Scott, 2005). ASA is deacetylated to salicylic acid (SA). SA is only to a minor extent hydroxylated by cytochrome P450 enzymes. The main way of metabolism of SA is the conversion to different types of glucuronides as performed by several UDP-glucuronosyltransferases (UGTs, e.g., UGT1A6) (Kuehl et al., 2006). Pharmacogenetic investigations in relation to ASA are rare. A genome-wide association study detected the polymorphism rs7572857G > A in the gene *CEP68*, encoding for a centrosomal protein of 68 kDa in length, to be a risk factor for aspirin intolerant asthma (Kim et al., 2010a) (Table 3). Using *in silico* tools for docking experiments, a research group predicted that the single-nucleotide polymorphism (SNP) *rs117122585* in the gene *PTGS1* encoding COX-1 might have the potential to decrease the inhibitory potential of ASA on COX-1 with putative consequences for adverse effect and efficacy potential of ASA in these patients (Marjan et al., 2014).

**TABLE 3:** Important pharmacogenetic markers in treatment of acute primary headaches.

Drug group	Example(s) of drug(s)	SNP	Therapeutic effect (1)	Effect on safety	Affected molecule
NSAIDs	Diclofenac	CYP2C8*3 and CYP2C9*2,*3	Higher effect in migraine Blanco et al. (2008), Dorado et al. (2008), Figueiras et al. (2016), Zajic et al. (2019)	Hepatotoxicity gastrointestinal bleeding Blanco et al. (2008), Figueiras et al. (2016), Zajic et al. (2019), McEvoy et al. (2021)	Lower activity of CYP2C8, CYP2C9 Blanco et al. (2008), Dorado et al. (2008), Figueiras et al. (2016), Zajic et al. (2019)
	Ibuprofen				
	Naproxen				
	Ketorolac				
	Ketoprofen				
—	—	PTGS2 *rs20417*	—	Rectal cancer risk Makar et al. (2013)	COX-2 Makar et al. (2013)
—	Diclofenac	UGT2B7*2	—	Hepatotoxicity gastrointestinal bleeding Daly et al. (2007)	Lower activity of UGT Daly et al. (2007)
Antiplatelet agents	Aspirin	*PTGS1 rs117122585*	Decrease or abolished effects Marjan et al. (2014) (in silico prediction)	—	COX-1 Marjan et al. (2014)
—	—	*CEP68 gene rs7572857G > A*	—	Increased risk for aspirin-associated asthma Kim et al. (2010a)	Centrosomal protein of 68 kDa in length Kim et al. (2010a)
Opioid analgesics	Tramadol	OCT1*3-*6	—	Higher risk for nausea, vomiting Tzvetkov et al. (2011)	OCT1 uptake strongly reduced or abolished Tzvetkov et al. (2011)
—	—	CYP2D6 gene duplication allele *1x2, *2x2, *35x2, *41x2 (UMs)	—	Sedation, nausea miosis increased risk for addiction Kirchheiner et al. (2008)	Higher activity of CYP2D6 Kirchheiner et al. (2008)
—	—	CYP2D6 *3, *4, *5, *6 (PMs)	Lower effect in migraine (Kirchheiner et al., 2008)	—	lower activity of CYP2D6 (Kirchheiner et al., 2008)
—	—	*OPRM1 (118A > G)*	Lower effect of pain therapy Stamer et al. (2016), (*in vivo* and *in vitro*)	Higher or lower doses of opioids may be needed to achieve same effect Stamer et al. (2016)	Changes in affinity of μ opioid receptor Stamer et al. (2016)
—	Butorphanol	*COMT rs4680*	Increase pressure pain threshold with *AG* genotype of *OPRM1 A118G* Mura et al. (2013)	—	changes in affinity of μ opioid receptor Mura et al. (2013)
Ergot derivatives	Ergotamine Dihydroergotamine	near *TSPAN2 rs12134493*	Lower effect in migraine without aura Moubarak and Rosenkrans, (2000)	Adverse effects not identified Moubarak and Rosenkrans, (2000)	TSPAN2 Moubarak and Rosenkrans (2000),Fang et al. (2018)
Triptans	Sumatriptan Zolmitriptan Eletriptan Naratriptan	*CYP1A2 -163A*	—	Higher risk of abuse Gentile et al. (2010)	Higher activity of CYP1A2 Gentile et al. (2010)
—	Triptans (unspecified)	*GNB3 gene C825CC*	Lower treatment effect in cluster headache Schürks et al. (2007),Gentile et al. (2010)	—	The G protein beta3 subunit Gentile et al. (2010)
—	Sumatriptan Zolmitriptan Eletriptan Naratriptan	*C939T*	Negative response to triptans in migraine Ishii et al. (2012)	—	DRD2 Ishii et al. (2012)
—	Eletriptan Rizatriptan Sumatriptan Frovatriptan Almotriptan Zolmitriptan	*SLC6A4 STin2 VNTR*	Inconsistent response to triptans in migraine Terrazzino et al. (2010)	—	Serotonin transporter Terrazzino et al. (2010)
—	Triptans (unspecified)s	*PRDM16 rs2651899*	Higher effect in migraine Christensen et al. (2016)	—	PR domain Christensen et al. (2016)

NSAIDs: Nonsteroidal anti-inflammatory drugs; CYP450: Cytochrome P; COX: Cyclooxygenase; UGT: Uridine 5′-diphospho-glucuronosyltransferase (UDP-glucuronosyltransferase); OCT1: Organic cation transporter 1; TSPAN2: tetraspanin 2; DRD2: dopamine receptor 2; TTH: Tension-type headache.

### Nonsteroidal Anti-Inflammatory Drugs

The analgesic and anti-inflammatory properties of NSAIDs lead to a wide use for the relief of acute migraine attacks and TTHs (Pavithra et al., 2020; Pardutz and Schoenen, 2010; National Clinical Guideline Centre (UK), 2012). The main mechanism of their action is the inhibition of the enzymes COX-1 and/or COX-2, which are responsible for the synthesis of prostaglandins that are involved in the inflammatory cascade and in hyperalgesia (Blanco et al., 2008; McEvoy et al., 2021). Adverse effects induced by nonselective NSAIDs are caused by the inhibition of constitutionally expressed COX-1 found in different tissues. The suppression of prostaglandin synthesis increases the risk for the development of upper gastrointestinal tract injuries and bleeding, renal fluid and natrium retention, and increased blood pressure (Pomes et al., 2019). NSAIDs are able to induce asthma due to a relative increase of leukotriene synthesis (Marjan et al., 2014). All NSAIDs are strongly metabolized in the liver and, thus, have a strong hepatotoxic potential (Table 1).

The metabolism of NSAIDs is realized in two stages. During phase one metabolism, NSAIDs are strongly metabolized by CYP2C8, CYP2C9, and CYP2C19 (Marjan et al., 2014). Phase two metabolism of NSAIDs is executed by different UDP-glucuronosyltransferases, such as the polymorphically expressed UGT2B7.

Genetic polymorphisms of the mentioned phase one genes, i.e., CYP2C8*3 and CYP2C9 2* and *3, are responsible for the reduced activity of the enzymes, which potentially lead to higher plasma levels of NSAIDs and, thus, stronger adverse effects of these drugs (Dorado et al., 2008; Figueiras et al., 2016; Zajic et al., 2019; Blanco et al., 2008). The mentioned genetic variants have been associated with an increased risk of adverse effects, including hepatotoxicity and gastrointestinal bleeding (Figueiras et al., 2016; Zajic et al., 2019; Blanco et al., 2008; McEvoy et al., 2021) (Table 3). Genetic polymorphisms in the genes coding for UDP-glucuronosyltransferases that are responsible for the conjugation of NSAID metabolites may also be associated with impaired clearance of NSAIDs. The polymorphism UGT2B7*2 is associated with reduced activity of the UDP-glucuronosyltransferase enzyme, which increases the risk of hepatotoxicity and damage to the upper gastrointestinal tract when using diclofenac (Daly et al., 2007). The polymorphism *rs20417* of the *PTGS2* gene encoding COX-2 has been associated with an increased risk of rectal cancer in case of chronic NSAID use in a population-based study of more than 3,000 colorectal cancer patients and healthy individuals in the USA (Makar et al., 2013).

### Opioid Analgesics

Opioids are rarely used for acute migraine due to their significant and undesirable adverse effects and the availability of safer and more effective drugs (Orr et al., 2015; Gong et al., 2014). However, butorphanol, meperidine, morphine, hydromorphone, and tramadol have been mentioned as beneficial agents for acute migraine treatment. Tramadol and butorphanol are the most commonly used compounds in this drug group in association with the mentioned indication. Butorphanol is an agonist–antagonist of opioid receptors (an agonist of κ-receptors and an antagonist of μ-receptors). Tramadol is an agonist of opioid receptors and also suppresses the reuptake of norepinephrine and 5-hydroxytryptamine (5-HT) in the central nervous system, thus, promoting hyperpolarization of nerve cell membranes with inhibition of nerve impulse conduction and enhancement of the activity of the antinociceptive system (Owusu Obeng et al., 2017; Stamer et al., 2016) (Table 1). The complex mechanism of action of tramadol allows affecting several key pathogenic features of migraine at once. The increase of monoamines affects the trigeminovascular system, and the activation of opioid receptors suppresses pain and negative emotional perception of pain. Tramadol induces opioid typical adverse effects, such as addiction, fatigue, weakness, lethargy, paradoxical stimulation, and sleep disorders (Kirchheiner et al., 2008; Tzvetkov, 2017). In addition to the central effects, tramadol can affect the gastrointestinal tract, inducing nausea, dry mouth, and constipation, the genitourinary system (difficulty urinating), and the cardiovascular system (arrhythmias and orthostatic hypotension) (Gong et al., 2014).

Tramadol is metabolized in two phases in the liver. Initially, CYP2D6 and CYP3A4 catalyze its N - and O-demethylation, followed by UGT1A8- and UGT2B7-dependent glucuronidation in the second phase (Tzvetkov, 2017). Polymorphisms in drug-metabolizing enzymes have a significant impact on tramadol pharmacokinetics and pharmacodynamics. Carriers of *CYP2D6* gene duplications (*1x2, *2x2, *35x2, and *41x2) belong to the group ultrarapid metabolizers (UMs) (Kirchheiner et al., 2008). The excessive concentration of the active metabolite O-desmethyltramadol in the plasma of UMs increases the risk of adverse effects with tramadol, such as sedation, nausea, miosis, and the development of addiction (Kirchheiner et al., 2008). On the contrary, carriers of combinations of the *CYP2D6* alleles*3, *4, *5, and *6, causing a poor metabolizer (PM) phenotype, are characterized by an increased concentration of tramadol in plasma decreased efficacy (Kirchheiner et al., 2008) (Table 3).

The organic cation transporter 1 accomplishes the uptake of tramadol and o-desmethyltramadol into the hepatocytes (Tzvetkov et al., 2011). As shown in postoperative patients, polymorphisms in the encoding gene *SLC22A1* [*rs12208357* (*3, Arg61Cy), *rs34130495* (*4, Gly401Ser)] greatly reduce the uptake and increase the risk of adverse effects (Tzvetkov et al., 2011). The polymorphisms *rs34059508* (*5, Gly465Arg) and *rs55918055* (*6, Cys88Arg) are associated with the complete absence of tramadol uptake by hepatocytes that further increase the risk of adverse events (Tzvetkov et al., 2011).

In the future, it may be of value to study the polymorphisms of *OPRM1* encoding the μ opioid receptor. Studies in animals and humans have shown that the *OPRM1* polymorphism 118A > G leads to interindividual differences in the sensitivity to pain and analgesic response to opioids and that carriers of the 118G allele may require higher opioid doses compared with carriers of the 118A allele (Stamer et al., 2016). As it was recently shown in a randomized placebo-controlled trial including 108 healthy individuals, the AA genotype of *rs4680* or *A_T_C_A/A_T_C_A* (*rs6269_rs4633_ rs4818_rs4680*) diplotype of *COMT* gene, combined with the *AG* genotype of *OPRM1 A118G*, showed significantly increased pressure pain threshold from butorphanol (Mura et al., 2013; Ho et al., 2020). However, studies investigating these SNPs specifically in migraine patients are missing.

### Ergot Derivatives

Ergot alkaloids are selective 5-HT1B, 5-HT1D, and possibly 5-HT1F receptor agonists (Diener and Limmroth, 2001). Ergotamine and dihydroergotamine have been on the market for a long time and are used to treat acute migraine and cluster headaches (Schiff, 2006) (Table 1). Ergotamine derivatives are substrates of CYP3A4 and should not be coadministered with strong CYP3A4 inhibitors (e.g., protease inhibitors, some macrolide antibiotics, and azole antifungals) due to the risk of acute ergot toxicity as shown in animal studies and humans (Moubarak and Rosenkrans, 2000; Srisuma et al., 2014). Pharmacogenetics has been poorly studied in association with these drugs. The presence of the tetraspanin 2 (*TSPAN2*) gene SNP *rs12134493* tended to be associated with a lower effect of ergotamine in migraine without aura, as studied in 244 Danish individuals (Christensen et al., 2016). This SNP has also been detected as a risk SNP for migraine in the Chinese Han population (Fang et al., 2018) (Table 3).

### Triptans

Triptans (e.g., sumatriptan, zolmitriptan, eletriptan, naratriptan, and rizatriptan) are used in acute migraine and cluster headaches. The main molecular targets of triptans are 5-HT1 receptors (Christensen et al., 2016; Ong and De Felice, 2018). Triptans induce cranial vasoconstriction (stimulation of 5-HT1B receptors), inhibition of the trigeminovascular system and neurovascular conflict, and the reduction of CGRP accumulation and release (stimulation of 5-HT1D receptors) (Ahn and Basbaum, 2005; Aggarwal et al., 2012) (Table 1). Additionally, triptans activate the antinociceptive system and stabilize trigeminal nucleus caudalis activity (Ahn and Basbaum, 2005). Triptans are contraindicated in patients with cardio- and cerebrovascular diseases, uncontrolled hypertension, and severe hepatic impairment and in patients with rare migraine variants such as hemiplegic migraine (Ahn and Basbaum, 2005).

Triptans are metabolized primarily by the monoamine oxidase A (MAO-A) system and by CYP1A2 and CYP3A4 (Aggarwal et al., 2012). In a study on 124 women, different MAO-A polymorphisms (leading to MAO-A-high or MAO-A-low activity) did not influence the efficacy or the risk of abuse of triptans to a significant extent (Gentile et al., 2010). At the same time, patients with the *-163A* allele polymorphism in *CYP1A2* have been shown to have higher enzyme activity and a higher risk of abuse compared with patients carrying the *-163C* allele (Gentile et al., 2010). In a study including 231 Caucasian patients, Schürks et al. (2007) demonstrated that a polymorphism in the gene encoding the G protein beta3 subunit (*GNB3 C825T*) is significantly associated with triptan treatment outcome in cluster headache (Gentile et al., 2010) (Table 3). The authors described a better response to treatment in the *GNB3 C825T* heterozygous group compared with carriers of the C*825C* genotype (Schürks et al., 2007; Gentile et al., 2010). Interestingly, carriers of the *C/C* genotype at the polymorphic position *C939T* in the *dopamine receptor 2 gene (DRD2)* were more likely to have a negative response to triptan treatment in migraine as investigated in a clinical trial including 46 consistent and 14 inconsistent responders to triptans in Japan (Ishii et al., 2012). The *STin2 VNTR* polymorphism in the gene *SLC6A4* encoding the serotonin transporter (SERT) was found to correlate with inconsistent response to triptans in migraine patients (Terrazzino et al., 2010). The *rs2651899* polymorphism in the *PRDM16* gene encoding the PR domain containing 16 proteins, a zinc finger transcription factor, appears to be associated with a better therapy efficacy of triptans in migraine, as shown in a Danish study including 1,806 patients, of which 1,386 reported an effect of triptans and 376 reported no effect (Christensen et al., 2016).

### Antidepressants

According to the recommendations of the EHF, antidepressants can be used for the preventive treatment of migraine and TTH. Amitriptyline and nortriptyline are used to prevent TTH, mirtazapine is a second-line drug, and venlafaxine is a third-line drug (Steiner et al., 2019). Amitriptyline has the best evidence for use in migraine prevention (Sarchielli et al., 2012; Burch, 2019b). Nortriptyline is an alternative in patients who may not tolerate amitriptyline (Burch, 2019b). Amitriptyline is mainly metabolized by CYP2C19 to active metabolites, including nortriptyline, and by CYP2D6, leading to a less active 10-hydroxy-metabolite (Dean et al., 2012a; Hicks et al., 2013). Amitriptyline blocks the uptake of serotonin and norepinephrine and has furthermore strong affinities for histamine (H1), alpha-1 adrenergic, and muscarinic (M1) receptors, which account for its adverse effects, such as sedation, weight gain, blurred vision, dry mouth, and constipation (UpToDate (2016). Tricycli, 2016). The main routes of nortriptyline metabolism are 10-hydroxylation (major route) and N-demethylation (a minor route) (Alexanderson and Borga, 1973; Mellström et al., 1981). 10-Hydroxylation is mediated mainly by cytochrome P450 2D6 (CYP2D6), although *in vitro* studies have shown that CYP3A4 is also involved (Venkatakrishnan et al., 19991999). Nortriptyline appears to be less toxic than other TCAs. It is, furthermore, the least problematic of the TCAs in drug interactions, being only a weak CYP2D6 and CYP2C19 inhibitor (Shin et al., 2002).

CYP2C19*17 creates a new binding site for the transcription factor that leads to higher expression and, thus, the activity of CYP2C19 (Sim et al., 2006). A trial including 50 patients with depressive disorders has observed that CYP2C19 UMs have a higher risk for adverse effects, such as, e.g., neuromuscular, anticholinergic/gastrointestinal, and symptoms, due to the accumulation of nortriptyline (Steimer et al., 2005). The Clinical Pharmacogenetics Implementation Consortium (CPIC) suggests monitoring CYP2C19 UMs with amitriptyline treatment tighter or choosing antidepressants with no major CYP2C19 metabolic pathway as an option (Hicks et al., 2013). However, it should be kept in mind that the frequency of alleles varies between different ethnic groups (Strom et al., 2012). Polymorphic variants associated with slow metabolism of CYP2C19, i.e., CYP2C19*2, *3, and *4, lead to a decrease in the metabolism of TCAs (Goldstein et al., 1997; Aynacioglu et al., 1999). These polymorphisms have been associated with a higher likelihood of adverse effects with TCAs, including anticholinergic adverse effects, cardiotoxicity, and dizziness (Dean et al., 2012a).

CYP2D6 UMs have an increased metabolism of TCAs, with reduced TCA plasma concentrations. CYP2D6*4/*41, *5/*9, and *4/*10, which encode for intermediate metabolizers (IMs), have a slightly reduced TCA metabolism with increased plasma concentrations and an increased risk for adverse effects, such as anticholinergic adverse effects, cardiotoxicity, and dizziness (Hicks et al., 2013). CYP2D6 PMs carrying variant combinations of CYP2D6*3, *4, *5, and *6 (Dean et al., 2012a) have the highest risk for adverse effects (Steimer et al., 2005). It is worth remembering that the frequency distributions of alleles vary between different populations (Sistonen et al., 2007).

A study of 23 women with chronic persistent midfacial TTH showed that carriers of the “LL” genotype of the serotonin transporter gene-linked polymorphic region (HTTLPR) responded better to the therapy with amitriptyline than patients with “SS” or “LS” genotypes (Agius and Muscat, 2019). A small trial in 80 patients detected a link between the *NOS3* gene Glu298Asp polymorphism, the production of NO, and the response of patients to TCAs when treated for migraine attacks. Migraine patients carrying the TT genotype produce less NO, which may lead to a better response to TCAs in comparison with carriers of GT and GG genotypes, which is reflected in a lower frequency of migraine attacks (Molana et al., 2014) (Table 4).

**TABLE 4:** Important pharmacokinetic markers in preventive treatment of primary headaches.

Drug group	Example(s) of drug(s)	SNP	Therapeutic effect	Effect on safety	Affected molecule
Antidepressants	Amitriptyline Nortriptyline	CYP2D6*4/*41, *5/*9, *4/*10 (IMs) CYP2D6*3-*4,*5, *6 (homozygous)	—	Higher risk for anticholinergic adverse effects, cardiotoxicity, dizziness Steimer et al. (2005)	Lower activity of CYP2D6 Steimer et al. (2005), Dean et al. (2012a), Hicks et al. (2013)
—	Amitriptyline	CYP2C19*2, *3, and *4	—	Higher risk of adverse effects, i.e., anticholinergic adverse effects, cardiotoxicity, dizziness Steimer et al. (2005), Dean et al. (2012a)	Lower activity of CYP2C19 Goldstein et al. (1997), Aynacioglu et al., 1999) higher activity of CYP2C19 Sim et al. (2006)
		CYP2C19*17			
——	TCAs	*NOS3*	Better treatment response in migraine Molana et al. (2014)	—	Lower activity of NOS3 Molana et al. (2014)
		*Glu298Asp*			
		*TT genotype*			
—	Amitriptyline	“LL” genotype of HTTLPR	Better treatment response in TTH Agius and Muscat, (2019)		HTTLPR Agius and Muscat, (2019)
—	Venlafaxine	*CYP2D6 (IMs and PMs)*	—	Gastrointestinal adverse effects such as nausea, vomiting and diarrhea Shams et al. (2006)	Lower activity of CYP2D6 Shams et al. (2006), Hicks et al. (2013)
		*HTR1B rs11568817*		Increased risk of bone complications Garfield et al. (2014), Rawson et al. (2017)	HTR1B Garfield et al. (2014), Rawson et al. (2017)
Calcium channel blockers	Verapamil	*PLCE1 rs10882386*	Higher effect in migraine Lopez et al. (2001), Smrcka et al. (2012), Cutrer et al. (2021)	—	PLC Lopez et al. (2001), Smrcka et al. (2012)
—	—	*ITGAL rs2230433*	Higher effect in migraine Cutrer et al. (2021)	—	Integrin alpha L chain of a heterodimeric integral membrane receptor protein Cutrer et al. (2021)
—	—	*EHBP1L1 rs6591182*	Higher effect in migraine Cutrer et al. (2021)	—	—
—	—	*STRIP2 rs676947*	—	—	—
		*SRP72 rs144645569*			
—	—	*CACNA1C 527974G > A rs2239050 CACNA1D rs312481G > A rs3774426C > T CACNB2 rs2357928*	—	Higher risk of cardiovascular outcomes Bremer et al. (2006), Kamide et al. (2009), Niu et al. (2010)	Voltage-gated calcium channel (VGCC) Bremer et al. (2006), Kamide et al. (2009), Niu et al. (2010)
ARBs	Candesartan	*LRP1 rs11172113*	Lower effect in migraine (trend) Christensen et al. (2016)	—	LRP1 Christensen et al. (2016)
—	—	*rs12134493*	Higher effect in migraine (trend) Christensen et al. (2016)	—	—
		*near TSPAN2*			
—	—	*rs6790925 near TGFBR2*	Higher effect in migraine (trend) Christensen et al. (2016)	—	—
—	—	*rs10504861 near MMP16*	Higher effect in migraine (trend) Christensen et al. (2016)	—	—
Anticonvulsants	Topiramate	*MDR1 C3435T*	Risk factor for treatment failure in migraine Atasayar et al. (2016)	—	MDR1 Atasayar et al. (2016)
—	Valproate sodium	*rs10504861 near MMP16*	Higher effect in migraine Christensen et al. (2016)	—	MMP16 Christensen et al. (2016)
—	—	*PRDM16 rs2651899*	Lower effects in migraine without aura Christensen et al. (2016)	Higher risk of hepatotoxicity Zhao et al. (2017)	PRDM16 Christensen et al. (2016)
		CYP2C9*3, CYP2A6*1/*4, CYP2A6*4/*4			CYP2C9, CYP2A6 Zhao et al. (2017)
		CYP2C19*2, CYP2C19*3		Higher risk of weight gain Noai et al. (2016)	CYP2C19 Noai et al. (2016)
		*UGT1A6 19T > G, 552A > C, 541A > G*		Ataxia, liver damage, metabolic changes, tremor, hallucinations, pancreatitis, and weight gain Goey et al. (2016)	Increased UGT enzyme activity Goey et al. (2016)
—	Phenytoin	CYP2C9*2	—	Higher likelihood skin and neurological adverse effects, hirsutism, teratogenesis (Mamiya et al., 1998, Ninomiya et al. (2000), Kidd et al. (2001), Ramasamy et al. (2007), Depondt et al. (2011), Babu et al. (2013), Dorado et al. (2013)	CYP2C9
		CYP2C9*3			—
		CYP2C19*2			CYP2C19
		CYP2C19*3			—
		CYP2C19*4			—
—	—	*EPHX1 113H 139R*	—	Teratogenesis, congenital craniofacial anomalies Dennery (2007) Azzato et al. (2010), Depondt et al. (2011), cutaneous adverse drug reactions: Stevens–Johnson syndrome and toxic epidermal necrolysis, hematologic and hepatic toxicity Karnes et al. (2021)	EPHX1
		HLA-B*15:02			HLA Karnes et al. (2021)
—	Carbamazepine Valproate sodium	GSTM1*0/*0	—	Carbamazepine and Valproate sodium induced hepatotoxicity Ueda et al. (2007), Fukushima et al. (2008), Chbili et al. (2018)	GSTM1 Bu et al. (2005)
—	—	*ABCC2 c. 1249 G > A*	—	Carbamazepine and Valproate sodium neurologic adverse effects Kim et al. (2010b)	MRP2,Kim et al. (2010b)
—	Carbamazepine	HLA-B*15:02, A*31:01, B*15:11,-A*02:01 B*57:01,-B*58:01	—	Higher risk for cutaneous adverse effects Hung et al. (2006), Man et al., 20072008, Ikeda et al. (2010), Kaniwa et al. (2010), McCormack et al. (2011)	HLA
		*EPHX1 C allele, C-G diplotype of 337T > C*		Stevens-Johnson syndrome and toxic epidermal necrolysis He et al. (2014)	EPHX1 He et al. (2014)
		CYP3A5*3/*3		CBZ toxicity Al-Gahtany et al. (2014)	CYP3A5 Al-Gahtany et al. (2014)
		CYP2C19*2 CYP2C19*3		Stevens-Johnson syndrome and toxic epidermal necrolysis Laska et al. (2017)	CYP2C19 Laska et al. (2017)
Neurotransmitter release antagonists	Onabotulinum toxinA	*TRPV1 rs222749 allele A*	Higher effect in migraine Moreno-Mayordomo et al. (2019)	—	TRP Moreno-Mayordomo et al. (2019)
—	—	*CALCA rs3781719 allele C*	—	—	CGRP Moreno-Mayordomo et al. (2019)

CYP450: Cytochrome P; TCAs: Tricyclic antidepressants; HTTLPR: serotonin transporter gene-linked polymorphic region; 5-HT: 5-hydroxytryptamine; LRP1: lipoprotein receptor-related protein 1; MMP16: matrix metalloproteinase-16; UGT: Uridine 5′-diphospho-glucuronosyltransferase (UDP-glucuronosyltransferase); EPHX1: Microsomal epoxide hydrolase; HLA: Human leukocyte antigen; GSTM1: Glutathione S-transferase class M1; TTH: Tension-type headache.

Mirtazapine, an atypical or tetracyclic antidepressant, is a noradrenergic and specific serotonergic antidepressant that increases central noradrenaline and serotonin by central presynaptic alpha‐2 antagonism; it also acts antagonistically on 5HT-2, 5HT‐3, and H1 receptors (Leung, 2021). Mirtazapine is metabolized in the liver by demethylation and then hydroxylation *via* cytochrome P450 enzymes (CYP1A2, CYP2D6, and CYP3A4) (Dahl et al., 1997). No studies were identified that have investigated genetic variants in relation to mirtazapine treatment outcome in headache disorders.

Venlafaxine belongs to the drug class of SNRIs. Venlafaxine is metabolized into the active metabolite, O-desmethylvenlafaxine, primarily by the CYP2D6 enzyme (Dean et al., 2015). Adverse effects occur more frequently in CYP2D6 PMs, such as vomiting and diarrhea, hypertension, tachycardia, and prolonged QTc interval (Johnson et al., 2006). Venlafaxine has been to a lesser extent investigated in pharmacogenetic studies than, for example, selective serotonin reuptake inhibitors, and the results of already performed investigations are, in some parts, contradictory or originate from single studies. However, it seems that several genetic polymorphisms are involved in its effectiveness and safety (Suwała et al., 2019). People with CYP2D6 *6/*4, *5/*4, or *6/*6 (PMs) are more likely to experience gastrointestinal adverse effects such as nausea, vomiting, and diarrhea (Shams et al., 2006). The *HTR1B* variant *rs11568817* seems to be a promising marker of an increased risk of bone complications during VEN treatment (Garfield et al., 2014; Rawson et al., 2017) (Table 4).

Duloxetine belongs to the drug class of SNRIs. CYP1A2 and CYP2D6 contribute predominantly to the metabolism of duloxetine (Kapur et al., 2014). In a clinical study of 45 people, duloxetine was shown to be effective in preventing episodic migraine (Young et al., 2013) and effective in a study investigating comorbid migraine and depression, including 30 individuals (Volpe, 2008). The administration of duloxetine to CYP2D6 PMs, along with inhibition of CYP1A2, led to clinically significant higher duloxetine exposure, emphasizing that coadministration with a potent CYP1A2 inhibitor should be avoided (Knezevic et al., 2018). No studies were identified regarding genetic variants and their impact on duloxetine efficacy in headache disorders.

### Calcium Channel Blockers

CCBs inhibit the inward movement of calcium by binding to the L-type “long-acting” voltage-gated calcium channels in the heart, vascular smooth muscle, and pancreas. Dihydropyridines belong to this group and act especially as peripheral vasodilators and are used for the treatment of hypertension, post-intracranial hemorrhage-associated vasospasm, and migraines (McKeever and Hamilton, 2021). The effect of CCBs in cluster headaches may be at least in part dependent on the reduction of calcium-dependent neurotransmission between climbing fibers and Purkinje cells in the cerebellum (Tso et al., 2021). CCBs are absorbed well orally, with a low bioavailability due to a high first-pass metabolism in the liver (Tso et al., 2021). Cinnarizine and flunarizine are metabolized mainly *via* CYP2D6 and CYP2B6 (Kariya et al., 1996). CYP450 3A4 and CYP1A2 are responsible for verapamil N-dealkylation, and the P450 2C subfamily (especially CYP2C8, CYP2C9) is responsible for verapamil O-dealkylation (Kroemer et al., 1993; Busse et al., 1995; Tracy et al., 1999).

Polymorphisms in several genes other than CYP genes have been recently associated with a decrease in the average duration of migraine when using verapamil, i.e., rs144645569 (*SRP72*), rs676947 (*STRIP2*), rs6591185 (*EHBP1L1*), and rs10882386 (*PLCE1*). In this study investigating the effect of migraine prophylaxis in 185 individuals using verapamil, the strongest decrease in the number of days with a headache after treatment was seen in carriers of the variant *rs2230433* in the *ITGAL* gene, which encodes the integrin alpha L chain of a heterodimeric integral membrane receptor protein that is involved in costimulatory signaling and intercellular adhesion (Cutrer et al., 2021). Also, significantly associated with headache reduction was *rs10882386* in *PLCE1*. *PLCE1* encodes a phospholipase C enzyme that catalyzes the hydrolysis of 1‐phosphatidyl‐1D‐myo‐inositol 4,5‐bisphosphate (Lopez et al., 2001; Smrcka et al., 2012). Several studies have shown that polymorphisms in genes encoding different ion channels, for example, voltage-gated calcium channel *CACNA1C (527974G > A and rs2239050)*, *CACNA1D (rs312481G > A and rs3774426C > T)*, and *CACNB2 (rs2357928)* influence the risk of adverse cardiovascular outcomes especially with verapamil (Bremer et al., 2006; Kamide et al., 2009; Niu et al., 2010) (Table 4).

### Angiotensin II Receptor Blockers and Angiotensin-Converting Enzyme Inhibitors

Angiotensin II receptor antagonists (or blockers, ARBs, candesartan) (Stovner et al., 2014; Sánchez-Rodríguez et al., 2021) and ACE inhibitors (lisinopril) can be used for the preventive treatment of migraine (Loder et al., 2012; Stovner et al., 2014; Steiner et al., 2019; Sánchez-Rodríguez et al., 2021). The main mechanism of action of ARBs is the blockade of angiotensin II type 1 (ATII) receptors, whereas the main mechanism of ACE inhibitors is the blockade of angiotensin I conversion to angiotensin II. ARBs and ACE inhibitors decrease angiotensin II-mediated vasoconstriction and aldosterone production, regulation of fluid and electrolyte balance, and regulation of morphological structure of vessels. The main area of their therapeutic use is the treatment of arterial hypertension and chronic heart failure (Tronvik et al., 2006; Do et al., 2014; Halker et al., 2016; Heidari et al., 2017). The mechanisms by which ARBs and ACE inhibitors act beneficially in migraine are not fully understood, but it is possible that their effectiveness is due to the blockade of type ATII receptors and decrease of the influence of angiotensin II in cerebral vessels, which reduces the tone of smooth muscles and increases their resistance to vasospastic stimuli; in addition, the decrease in aldosterone production under the action of ARBs may play a role (Tronvik et al., 2006). Serious adverse effects of ARBs occur infrequently; sometimes, there is slight dizziness, less often headache, as well as an increase in the frequency of respiratory infections and also includes dry cough for ACE inhibitors (Lopez et al., 2001; Cutrer et al., 2021). ARBs are metabolized in the liver, but the main part is excreted unchanged by the kidneys, whereas lisinopril is fully excreted unchanged by the kidneys.

In migraineurs, only nominal significant associations between a polymorphism in *LRP1 (rs 11172113)* encoding the low-density lipoprotein receptor-related protein 1 (lower efficacy), *rs12134493* near the tetraspanin 2 gene *TSPAN2*, and *rs6790925* near the transforming growth factor, beta receptor II gene *TGFBR2* and *rs10504861* near the matrix metalloproteinase-16 gene *MMP16* (better efficacy) have been found for migraine patients treated with sartans (Christensen et al., 2016). There is no evidence that polymorphisms in the RAS system are important for the efficacy and safety of lisinopril or ARBs in migraineurs (Tronvik et al., 2008).

### Anticonvulsants

Valproate sodium, topiramate, phenytoin, and carbamazepine (CBZ) are used to treat migraine (Christensen et al., 2016; Atasayar et al., 2016; Fricke-Galindo et al., 2018a). Topiramate is also efficacious in cluster headache and may have some effect in migraine with medication overuse (Diener et al., 2007; Silberstein et al., 2007; Diener et al., 2009; Steiner et al., 2019) (Table 2).

Valproic acid increases the level of gamma-aminobutyric acid (GABA) by inhibiting the GABA-transaminase, which is involved in the metabolism of the transmitter (Ghodke-Puranik et al., 2013). It also blocks voltage-gated sodium, potassium, and calcium channels (including those coded for by *CACNA1C*, *CACNA1D*, *CACNA1N*, and *CACNA1F*) and the gene family encoding voltage-gated sodium channels (SCN) (Johannessen and Johannessen, 2003; Van den Berg et al., 1993). Valproic acid is 50% glucuronidated and 40% β-oxidized in the mitochondria. It is to a minor extent metabolized by cytochrome P450 (CYP)-mediated oxidation (10%) (Ito et al., 1990; Argikar and Remmel, 2009; Tan et al., 2010). Carriers of the variants CYP2C9*3 and CYP2A6*4 showed increased levels of hepatotoxic metabolites compared with the wild type, as shown in 279 individuals from China (Zhao et al., 2017). Mutations in a DNA polymerase subunit gamma (POLG) gene are also related to increased incidence of serious liver adverse effects in patients under valproate therapy, as shown in a study including 17 individuals from the USA (Stewart et al., 2010). Japanese females carrying the variants CYP2C19*2, or CYP2C19*3, were more susceptible to valproate-induced weight gain (Noai et al., 2016). Carriers of *UGT1A6 19T > G*, *552A > C*, and *541A > G* alleles displayed increased UGT enzyme activity compared with wild-type carriers, whereas *UGT1A6 552A > C* carriers showed a longer elimination half-life and a lower clearance rate associated with VPA-related adverse drug reactions, such as ataxia, liver damage, metabolic changes, tremor, hallucinations, pancreatitis, and weight gain (Goey et al., 2016) (Table 4). There were no significant correlations between the treatment responses to valproic acid and *MDR1*, *CYP2D6*, and *CYP2C19* gene polymorphisms, as shown in the study, including 251 migraine patients (230 female and 21 male subjects) with or without aura (Atasayar et al., 2016). Based on data from the Danish Headache Centre, the effectiveness of anticonvulsant treatment in all types of migraine in humans is increased in the case of polymorphism *rs10504861* near *MMP16*, whereas in the case of *PRDM16* variant *rs2651899*, the effectiveness of anticonvulsant treatment of migraine without aura is reduced as investigated in 365 migraine cases (Christensen et al., 2016).

The mechanism of action of CBZ is not well understood, but it has been suggested that it binds mainly to sodium channels and also interacts with calcium and potassium channels. CBZ is a positive allosteric modulator of GABA-A receptor (Iannaccone et al., 2021). CBZ is almost entirely metabolized in the liver by epoxidation and hydroxylation (Fricke-Galindo et al., 2018b). CBZ-10,11-epoxide is formed by CYP3A4, CYP3A5, and CYP2C8. This reactive metabolite is pharmacologically active and potentially toxic (Korinthenberg et al., 1994) due to its capability to form covalent protein adducts (Bu et al., 2005). CBZ-10,11-epoxide is further metabolized to the inactive molecule by the microsomal epoxide hydrolase (EPHX1) (Pearce et al., 2002). Both glutathione S-transferase class M1 (GSTM1) and class T1 (GSTT1) are involved in conjugation reactions of the main metabolite and also in the detoxification of toxic metabolites of CBZ (Bu et al., 2005). Findings in 129 Tunisian epileptic patients treated with CBZ suggest that the GSTM1 (-) allele may be considered as a risk factor for CBZ-induced hepatotoxicity (Chbili et al., 2018). Japanese researchers observed a link between the lack of GSTM1 and increased levels of hepatic enzymes in 192 patients treated with CBZ and in 149 patients treated with valproate sodium (Ueda et al., 2007; Fukushima et al., 2008). The polymorphism c. 1249 G > A in *ABCC2* encoding for the transporter MRP2 has been linked with neurological adverse reactions in patients using CBZ and valproic acid (Kim et al., 2010b) (Table 4).

The *EPHX1* gene polymorphism 337T > C has been associated with an increased risk for adverse effects such as Stevens–Johnson syndrome and toxic epidermal necrolysis in patients with epilepsy, putatively through an increase in the concentration of a CBZ 10,11-epoxide (He et al., 2014). CBZ toxicity has been furthermore more often observed in patients carrying CYP3A5*3/*3 (Al-Gahtany et al., 2014), CYP2C19*2, and CYP2C19*3 (Laska et al., 2017) (Table 4).

Genes encoding the human leukocyte antigens (HLAs) are organized as a complex on chromosome 6. They encode cell-surface proteins that regulate the actions of the immune system (Barquera et al., 2008). Certain HLA alleles (B*15:02, A*31:01, B*15:11, A*02:01, B*57:01, and B*58:01) have been associated with a higher risk for cutaneous adverse effects in people of different nationalities when using CBZ (Hung et al., 2006; Man et al., 20072008; Ikeda et al., 2010; Kaniwa et al., 2010; McCormack et al., 2011). The Clinical Pharmacogenetics Implementation Consortium recommends genotyping for HLA-B*15:02 and HLA-A*31:01 alleles (Phillips et al., 2018). This information is furthermore included in the CBZ label of FDA and the European Medicines Agency, i.e., recommending genetic testing for HLA gene polymorphisms (US Food and Drug Administration, 2007) (Table 4).

Phenytoin inhibits voltage-gated sodium channels (Yang et al., 2012). The drug has a hepatotoxic potential (FDA, 2020; Karnes et al., 2021). Phenytoin metabolism is almost completely hepatic. CYP2C9 is responsible for 90% of metabolism, and CYP2C19 is responsible for the remaining 10%. Studies in patients have shown that carriers of the variants CYP2C9*2, CYP2C9*3 and CYP2C19*2, CYP2C19*3, CYP2C19*4, show a higher likelihood for adverse effects with phenytoin, such as gum hyperplasia, hirsutism, skin and neurological symptoms, such as confusion, dysarthria, memory loss or deterioration, astasia, and dizziness (Mamiya et al., 1998; Ninomiya et al., 2000; Kidd et al., 2001; Ramasamy et al., 2007; Depondt et al., 2011; Babu et al., 2013; Dorado et al., 2013). EPHX1 produces reactive oxide intermediates, which causes the teratogenic phenytoin effect (Dennery, 2007; Depondt et al., 2011). The genetic variants 113H and 139R of EPHX1 have been associated with congenital craniofacial anomalies in newborns in connection to phenytoin treatment (Azzato et al., 2010). The HLA-B*15:02 allele has been associated with phenytoin-induced cutaneous adverse drug reactions, including Stevens–Johnson syndrome and toxic epidermal necrolysis (Table 4).

Topiramate modulates voltage-activated sodium channels and cation influx through α-amino-3-hydroxy-5-methyl-4-isoxazole propionic acid and kainic acid receptor channels, potentiates GABAA receptor-mediated currents, and inhibits carbonic anhydrase isoenzymes (Zona et al., 1997; Poulsen et al., 2004; Gryder and Rogawski, 2003). Topiramate is rapidly and completely absorbed after oral administration (Fan et al., 2016). Protein binding is at a 3–4% low (Doose et al., 1996). Eighty-five percent of the dose is excreted unchanged in the urine (Garnett, 2000), and the remaining 15% is metabolized through hydrolysis, hydroxylation, and glucuronidation (Johannessen, 1997). Topiramate is exported out of the brain tissue by MDR1 (Wang-Tilz et al., 2006). According to a study performed in 251 patients at a headache outpatient clinic in Turkey, topiramate is more efficient against migraine in carriers of the *MDR1* genotype *3435TT* compared with carriers of the genotypes CC and CT. The *ABCB1* polymorphism *C3435T* was also found to be a risk factor for topiramate treatment failure in migraine as studied by Atasayar et al. (2016) in a clinical trial including 251 individuals (Table 4). There was no significant relationship between treatment response to topiramate in migraine patients and either *CYP2D6* or *CYP2C19* polymorphisms (Atasayar et al., 2016).

### Ditans, Gepants, and Anti-Calcitonin-Gene-Related Peptide Monoclonal Antibodies

In recent years, new classes of drugs have been approved for the treatment of migraine, whose mechanisms of action are associated with specific effects on the pathophysiology of migraine (Szkutnik-Fiedler, 2020). These include ditans, gepants, and anti-CGRP monoclonal antibodies (Negro and Martelletti, 2019; Szkutnik-Fiedler, 2020). Ditans (e.g., lasmiditan) selectively activate 5-HT_1F_ receptors, which determines their effectiveness similar to triptans and leads to a decrease of CGRP release, with fewer adverse effects due to vasoconstriction, as ditans do not bind to 5-HT_1B_ and 5-HT_1D_ receptors (Stewart et al., 2010). Gepants (telcagepant, ubrogepant, rimegepant, and atogepant) act directly by blocking CGRP receptors and reduce vascular nociceptive transmission and trigeminal nociceptive activation (Garnett, 2000). Anti-CGRP monoclonal antibodies have a similar effect. Galcanezumab, fremanezumab, and eptinezumab bind CGRP directly with high affinity, whereas erenumab binds to the CGRP receptor and blocks its CGRP binding (Garnett, 2000). There are currently no studies on the pharmacogenetics of these drugs and no data available on gene polymorphisms that could affect their efficacy and safety (Johannessen, 1997). It can be hypothesized that various polymorphisms in the 5-HT_1F_ receptor system and the relating signaling pathways may be associated with the efficacy of ditans. It is possible that polymorphisms in the CGRP system and its receptor are most closely associated with the efficacy and safety of gepants and anti-CGRP monoclonal antibodies (Johannessen, 1997). Further studies on the pharmacogenetics of ditans, gepants, and anti-CGRP monoclonal antibodies are needed to elucidate polymorphisms that are important for their efficacy and safety.

### Corticosteroids

Blumenthal (1952) and Frohner (1953) were the first to demonstrate a clinical effect of corticosteroids in migraine. Corticosteroids reduce pain by inhibiting prostaglandin synthesis, which leads to inflammation, and by reducing vascular permeability that results in tissue edema (Watanabe and Bruera, 1994; Mensah-Nyagan et al., 2009). Exogenous administration of corticosteroids suppresses excessive stress and inflammatory response from the hypothalamic–pituitary axis (Chrousos and Gold, 1992). Steroid receptors are found in the central and peripheral nervous systems and are responsible for the growth, differentiation, development, and plasticity of neurons (Watanabe and Bruera, 1994). In particular, corticosteroids reduce spontaneous discharge in an injured nerve, which reduces neuropathic pain (Mensah-Nyagan et al., 2009). Corticosteroids suppress neurogenic inflammation (Mensah-Nyagan et al., 2009) and modulate neuroplasticity, thereby reducing pain (Mensah-Nyagan et al., 2009). In the literature, single-dose intravenous dexamethasone has been discussed as a treatment option for managing resistant, severe, or prolonged migraine attacks (Woldeamanuel et al., 2015). Corticosteroids are not recommended by EHF and AAN for migraine treatment (Loder et al., 2012). A suboccipital steroid injection can be used as a preventive treatment for episodic and chronic cluster headaches. Greater occipital nerve blockade is highly effective in reducing headache attacks and even aborting cluster bouts in cluster headache patients without requiring additional prophylactic treatment, which has been proven in experiments involving 51 and 60 patients (Gantenbein et al., 2012; Gönen et al., 2019). At present, no studies have been found investigating the pharmacogenetics of these drugs in an association of drug effectiveness in cluster headaches.

### Benzodiazepines

The actions of benzodiazepines are due to the potentiation of the neural inhibition that is mediated by GABA. Practically all effects of the benzodiazepines result from their actions on the ionotropic GABA (A) receptors in the central nervous system. Benzodiazepines do not activate GABA (A) receptors directly, but they require GABA. The main effects of benzodiazepines are sedation, hypnosis, decreased anxiety, anterograde amnesia, centrally mediated muscle relaxation, and anticonvulsant activity (Möhler et al., 2002).

EHF, AHS, and AAN do not list the use of benzodiazepines in their guidelines for preventive or acute therapy of migraine, TTH, or cluster headache (Loder et al., 2012; Silberstein et al., 2012; Marmura et al., 2015; Robbins et al., 2016; Steiner et al., 2019). Benzodiazepines are sometimes used in clinical practice for the treatment of different headache types such as migraines, TTH, or medication overuse headache and are therefore mentioned here.

Diazepam is metabolized in the liver with only traces of the unchanged drug being excreted in the urine. The two major pathways of diazepam metabolism, the formation of N-desmethyldiazepam and temazepam, are catalyzed by different CYP isoforms (Inaba et al., 1988). The third potential metabolite, 4-hydroxydiazepam, seems to be less important. Studies with a series of CYP isoform-selective inhibitors and an inhibitory anti-CYP2C antibody indicate that temazepam formation is carried out mainly by CYP3A isoforms, whereas the formation of N-desmethyldiazepam is mediated by both CYP3A isoenzymes and CYP2C19 (Andersson et al., 1994; Kato and Yamazoe, 1994).

Benzodiazepines have adverse effects, including dependence, rebound anxiety, memory impairment, discontinuation syndrome, and others and should therefore be used with caution and only over a short time (Uzun et al., 2010). A clinical trial including 144 Japanese individuals tested a combined treatment of TTH with etizolam, a benzodiazepine derivative, and NSAIDs and showed that this treatment is effective in young patients (Hirata et al., 2007).

In a study including 30 Russian individuals, it was observed that CYP2C19*1/*2 and CYP2C19*2/*2 genotypes increase the risk of adverse effects when using diazepam, whereas CYP2C19*1/*17 and CYP2C19*17/*17 genotypes, on the contrary, reduce the risk of adverse effects, which was later confirmed in a second trial including 50 Russian subjects (Skryabin et al., 2020; Skryabin et al., 2021). Similar observations were made in other populations (Bertilsson et al., 1989; Wan et al., 1996; Qin et al., 1999; Dean et al., 2012b). In 3,705 elderly patients, CYP2C9*2 and CYP2C9*3 were shown to be associated with an increased fall risk (Ham et al., 2017). The effect of the polymorphism of CYP3A5 on the pharmacokinetics of benzodiazepines remains inconclusive, as some studies show a negligible effect on the pharmacokinetics of drugs (Kuehl et al., 2001; Shih and Huang, 2002; Park et al., 2006).

### OnabotulinumtoxinA

OnabotulinumtoxinA has demonstrated high efficacy and is approved for the treatment of chronic migraine (Silberstein et al., 2012; Steiner et al., 2019). This drug is a toxin of *Clostridium botulinum*, which disrupts the release of neurotransmitters from the presynaptic membrane into the synaptic cleft (Affatato et al., 2021). Currently, its mechanisms of action in migraine are not fully understood. There is evidence that onabotulinumtoxinA disrupts the release of glutamate, substance P, and CGRP in the trigeminally innervated craniofacial–cervical region, which blocks key mechanisms of migraine attack pathophysiology (Szok et al., 2015; Burstein et al., 2020; Affatato et al., 2021). In the context of beneficial effects, specifically in chronic migraine, the ability to reduce central sensitization under the action of onabotulinumtoxinA has to be mentioned, which underlies the reduction in the frequency and intensity of migraine attacks (Szok et al., 2015; Burstein et al., 2020; Affatato et al., 2021).

There are currently no reliable data on the pharmacokinetics and metabolism of onabotulinumtoxinA due to its high neurotoxicity, but it is most likely that most of the toxin is cleaved by proteases, and products of proteolysis are used in normal metabolic pathways (Frampton and Silberstein, 2018). OnabotulinumtoxinA is determined in negligible concentrations in plasma and has low immunogenicity, but it has been shown that toxin-neutralizing antibodies can be synthesized after 12 weeks of its use (Frampton and Silberstein, 2018).

Two SNPs have been associated with onabotulinumtoxinA efficacy in chronic migraine. R*rs222749* located on the gene *transient receptor potential cation channel subfamily v member 1* (*TRPV1*) occurred with a frequency of 12.5% in nonresponders and 4.17% in the group of responders and SNP *rs3781719* of the gene *calcitonin related polypeptide alph*a (*CALCA*) where allele C represents 26.9% in responders and 40.9% in nonresponders (Moreno-Mayordomo et al., 2019) (Table 4). Further studies of genetic polymorphisms associated with the efficacy and safety of OnabotulinumtoxinA in the treatment of chronic migraine are needed.

## Discussion

The review provides a comprehensive overview regarding the pathophysiology, therapy safety, and efficacy of three important primary headache disorders, i.e., TTH, migraine, and cluster headache. In this context, the review summarizes the current options regarding pharmacological treatment of acute and chronic forms of the mentioned headache types and, most importantly, sheds light on the current state of the art regarding the impact of pharmacogenetic variants on the safety and efficacy of compounds used in the acute and preventive treatment of migraine, TTH, or cluster headache.

Genetic polymorphisms of relevance identified in the area of headache disorders are foremost associated with a change in the risk for adverse effects of the therapeutics used. This includes several variants in genes that are responsible for phase I and phase II metabolism or transport of the therapeutics discussed. Important examples comprise CYP genes such as *CYP2C8*, *CYP2C9* (CYP2C8*3, CYP2C9*2, and *3), variants in *CYP2D6* (PM variants such as, e.g., *3 -*6 or gene duplications/multiplications), and in *CYP2C19* (PM variants *2, *3, UM variant *17). Variants in these genes have been shown to modify the safety of compounds belonging to often used drugs classes NSAIDs (*CYP2C8*, *CYP2C9*), antiepileptics (*CYP2C9*, *CY2C19*, phenytoin), or antidepressants (*CYP2D6*, *CYP2C19*, TCAs) and to more seldomly used drug classes such as opioids (*CYP2D6*). Moreover, polymorphisms in genes encoding for enzymes involved in phase II metabolism, such as in UDP-glucanosyltransferases [e.g., UGT2B7*2 (NSAIDs)] and GSTM1 (GSTM1 *0/*0, CBZ, valproate sodium), as well as SNPs in drug transporters such as MDR1 (ABCB1, opioids, topiramate) organic cation transporter 1 (SLC22A1, opioids) or MRP2 (ABCC2, CBZ), have been associated with modulations of drug safety in headache disorders. SNPs in pharmacodynamic targets have been to a lesser extent linked with side drug adverse effects in headaches, but examples of such variants can be found in the COX 1 gene (*PTGS1*; aspirin) or in *OPRM1* (opioids) or in voltage-gated calcium channels (verapamil). Only a few examples could be found, where genes with functions other than pharmacokinetics or -dynamics have been linked to drug safety in headache disorders. An important example displays variants in the HLA system that have been associated with partly severe adverse effects of phenytoin and CBZ. Another example comprises the polymorphically expressed gene *CEP68* that has been associated with aspirin-induced asthma.

The number of studies investigating genetic variants in relation to the therapy success of headache therapeutics is much smaller. Those studies include only small sample sizes, which lead to overall much less well characterized genetic variants associated with therapeutic efficacy in headache disorders as compared with SNPs linked to drug safety in this therapeutic area. It also becomes obvious that studies investigating pharmacogenetics in relation to therapy efficacy have so far mainly concentrated on migraine, whereas efficacy-related pharmacogenetics studies investigating cluster headache or TTH patients have only very rarely been performed. Interestingly, variants associated with drug efficacy show a much more heterogeneous gene spectrum compared with polymorphically expressed genes that have been linked to drug safety. Genetic variants in genes such as *SLC6A4* (SERT), *DRD2* (dopamine receptor), *PRDM16* or *CYP1A2*, and *GNB3* have been associated with a modified therapy outcome with triptans in migraine and cluster headache, respectively. Of note, a polymorphic expression of PRDM16 has been, together with MMP16, also linked to modulated therapy success with anticonvulsants in migraine. Furthermore, genetic variants related to *MMP16*, *TGFBR2*, *TSPAN2*, and *LMP1* have been linked to therapy outcome changes with sartans in migraine. In this context, it is noted that polymorphisms in genes including *TGFBR2*, *TSPAN2*, and *PRDM16* are also associated with migraine risk (Gormley et al., 2016), underlining a potentially important role of these genes in migraine pathogenesis and therapy success in migraine. Likewise, MMP16 should be further investigated with regard to efficacy shifts due to the repeatedly observed influence on therapy effect with different compounds. Further pharmacogenetics studies with larger sample sizes are needed to confirm the described findings and observations. The knowledge regarding polymorphisms and their impact on therapy outcomes in headache disorders is very limited for opioids and TCAs. Likewise, pharmacogenetic markers for therapy efficacy in headache disorders when using ergot derivatives, beta-blockers, and novel therapy options such as ditans, anti-CGRP monoclonal antibodies, and gepants have not been studied enough to be able to draw conclusions on the usability of genetic variants for therapy response prediction in headache disorders.

Although a limitation of this review may be that the search was not performed following a systematic protocol such as PRISMA, the review is, to our knowledge, unique in the sense that it very comprehensively sheds light on both the safety and efficacy of acute and preventively used drugs of three important headache disorders taking established and novel knowledge on pharmacogenetic markers into consideration.

The main goal of precision medicine is to prescribe the most appropriate, effective, and safe treatment for the disease based on the individual characteristics of the patient (including genetic polymorphisms of enzymes significant for the pharmacodynamics and pharmacokinetics of drugs). A wide class of medications is used in the treatment of headaches, for both the relief of attacks and the prevention of chronic headaches, which have a wide range of adverse effects (Pomes et al., 2018), as presented in this review. Additionally, there is an increased risk of life-threatening drug-to-drug interactions when they are used in combination, especially in comorbid patients who are being treated for multiple diseases simultaneously, as summarized in Pomes et al. (2018). It can be concluded that there is currently not enough data available to be able to create a unified system and concept for the selection of headache drugs for a particular patient with headaches only based on his genetic profile (Cader, 2020). Although advances have been made regarding an understanding of genetic risk factors important for adverse effects of compounds used for migraine, TTH, or cluster headache, it becomes obvious that more pharmacogenetics studies are needed exploring the influence of genetic variants on therapy outcome in primary headache disorders. This is especially relevant for novel therapeutic options such as ditans, CGRP monoclonal antibodies, and gepants, for which studies regarding genetic variants and their impact on efficacy are currently missing. In this context, pharmacogenetic effect studies in migraine may also further explore polymorphically expressed genes that have been identified to influence both disease risk and therapy response to further understand the importance of these genes for individual disease risk and therapy adaptation.

## Footnotes

1
https://www.clinicaltrialsarena.com/projects/aimovig-erenumab-treatment-episodic-migraine/

## Glossary

5-HT: 5-hydroxytryptamine
5-HTR: 5-HT receptors
AAN: American Academy of Neurology
ACE inhibitors: Angiotensin converting enzyme inhibitors
ARBs: Angiotensin II type 1 receptor blockers
CBZ: Carbamazepine
CCB: Calcium channel blockers
CGRP: Calcitonin-gene related peptide
COX: Cyclooxygenase
CYP450: Cytochrome P450
DPWG: Dutch Pharmacogenetics Working Group
EHF: European Headache Federation
EPHX1: Microsomal epoxide hydrolase
FDA: Food and Drug Administration
GABA: γ-aminobutyric acid
GFR: Glomerular filtration rate
GSTM1: Glutathione S-transferase class M1 GSTT1: Glutathione S-transferase class T1
H1 receptors: Histamine receptors type 1
HLA: Human Leukocyte Antigen
IM: Intermediate metabolizer
MAO: Monoamine oxidase
MAT: Monoamine transporter
NSAIDs: Nonsteroidal anti-inflammatory drugs
OPRM1: Opioid receptor μ
RAMP1: Receptor activity-modifying protein 1
SERT (5-HTT): Serotonin (5-HT) transporter
SNRIs: Serotonin–norepinephrine reuptake inhibitors
TCAs: Tricyclic antidepressants
UGT: Uridine 5′-diphospho-glucuronosyltransferase (UDP-glucuronosyltransferase)
